# Effect of High-Pressure Processing on Fresh Sea Urchin Gonads in Terms of Shelf Life, Chemical Composition, and Microbiological Properties

**DOI:** 10.3390/foods11030260

**Published:** 2022-01-19

**Authors:** Valentina Coroneo, Francesco Corrias, Andrea Brutti, Piero Addis, Efisio Scano, Alberto Angioni

**Affiliations:** 1 Department of Medical Science and Public Health, Food Hygiene Laboratory, University Campus of Monserrato, University of Cagliari, SS 554, 09042 Cagliari, Italy; coroneo@unica.it; 2 Food Toxicology Unit, Department of Life and Environmental Science, University Campus of Monserrato, University of Cagliari, SS 554, 09042 Cagliari, Italy; francesco.corrias@unica.it (F.C.); addisp@unica.it (P.A.); 3 Experimental Station for the Food Preservation Industry—Research Foundation, Viale Tanara 31/a, 43121 Parma, Italy; andrea.brutti@ssica.it; 4 Faculty of Agraria, University of Sassari, Viale Italia 39/a, 07100 Sassari, Italy; efisiscano@gmail.com

**Keywords:** sea urchin, HPP, microbial analysis, fatty acid, amino acid

## Abstract

*Paracentrotus lividus* is a widespread sea urchin species appreciated worldwide for the taste of its fresh gonads. High-pressure processing (HPP) can provide a thermal equivalent to pasteurization, maintaining the organoleptic properties of the raw gonads. This study evaluated HPP technology’s effect at 350 MPa and 500 MPa on microbial inactivation and biochemical characteristics of *P. lividus* gonads. HPP at 350 MPa resulted in a higher decrease in protein and free amino acids associated with a loss of olfactory, color, and gustatory traits and a visual alteration of the texture. On the other hand, gonad samples stored for 40 days after treatments at 500 MPa showed a good organoleptic profile similar to fresh gonads. Furthermore, only 500 MPa effectively reduced mesophilic bacteria contamination among the two HPP treatments carried out. Total lipids increased during storage; however, the SAFA/PUFA rate was homogeneous during HPP trials ranging from 2.61–3.91 g/100 g. Total protein decreased more than 40% after HPP at 350 MPa, whereas, after 500 MPa, it remained stable for 20 days. The amount of free amino acid constantly decreased during storage after HPP at 350 MPa and remained constant at 500 MPa. HPP can effectively remove the bacterial flora and inactivate enzymes, maintaining the properties of the fresh sea urchin gonads.

## 1. Introduction

*Paracentrotus lividus* (Lamark 1816) is a widespread sea urchin species in the Mediterranean area, appreciated worldwide for the taste of its gonads [1]. *P. lividus* is a sedentary echinoderm that mainly colonizes rocky bottoms rich in vegetation or lives on sandy bottom colonies within the *Poseidonia oceanica*. *P. lividus* feeds on plant forms and animal organisms such as small crustaceans and mollusks, sponges, and coelenterates [2]. The edible part is represented by the reproductive system, the gonads, commonly called pulp or eggs.

Sea urchin gonads have high water and protein levels and low lipid and carbohydrate content and can be considered a low-calorie commodity [3].

The fat fraction is characterized by significant polyunsaturated fatty acids (PUFAs). Eicosapentaenoic acid (EPA), docosahexaenoic acid (DHA), docosapentaenoic acid (DPA), docosatetraenoic acid, and docosadienoic acid are the most represented [4].

The commercial value of the gonads is strictly associated with their organoleptic features, which consists of maintaining their granular surface without melting. Consumers demand a product that is easy and ready to use, easy to store, and economical, with a high degree of food safety, and a high nutritional and organoleptic value. Therefore, food preservation approaches should respect all quality parameters requested for sea urchins. Gonads can be commercialized fresh, frozen, and sterilized after packaging in tinplate boxes and glass jars. Fresh gonads recovered from the shell (consisting of calcite which incorporates various metals), stored at 5 °C, and with an expected shelf life of about 5 days are intended for immediate consumption and local market only [5]. However, this preparation can present microbiological problems of contamination. The freezing process, conducted at −30/−40 °C, or more often at −18 °C, decreases the enzymatic and microbial activity allowing storage up to 3 months before flavor and texture lessening [5].

High-temperature food preservation technologies (110 °C for 90 s) are known to denature proteins and lower fatty-acid content in food [6], in addition to changing the rheologic properties and modifying sea urchin nutritional and organoleptic characteristics. For this reason, the decontamination systems based on heat do not apply to *P. lividus*.

High-pressure processing (HPP) is classified as a nonthermal method of preservation; for this reason, it has been accepted and adopted for commercial processing on many foods worldwide. HPP can provide a thermal equivalent to pasteurization, ensuring microbial safety without changing the raw material’s color, flavor, and texture during storage [7].

The antimicrobial mechanism of HPP consists of an alteration of macromolecules, intracellular proteins, hydrophobic bonds, and electrostatic interactions weakening cell membranes and inducing cell death. HPP deactivation is affected by microbial species, pressure level, and holding time applied to the food product’s physicochemical characteristics (aw and pH) [8,9,10,11,12,13,14]. Low pH values showed a higher degree of microbial inactivation [8]. However, HPP applications for viruses and bacteria should consider the individual characteristics of the pathogenic microorganisms; hepatitis (HAV) can be reduced between 300 and 450 MPa, whereas poliovirus needs 5 min at 600 MPa, and norovirus needs 5 min at 275 MPa [15,16].

Although lethal to microorganisms, pressure treatment does not break covalent bonds, showing a minimal effect on food nutritional and sensory properties. Moreover, this process’s low energy value supply does not promote the development of undesirable chemical compounds, such as free radicals, that could rise with other preservation methods. Thus, the final sanitized product shows organoleptic and nutritional characteristics comparable to the fresh product [8].

Today, the use of HPP is extensively diffused in the food industry, and the commercialization of pressurized foods has increased around the world to produce jams, juices, sauces, smoothies, ready-to-eat meat products, guacamole, and oysters [11,17,18].

Numerous studies have been performed on fish, especially cod, salmon, and mackerel. Studies have highlighted that different species behave differently depending on the species and HPP setting; moreover, the level of fats and protein can be influenced, leading to nutritional modifications [19,20]. In addition, fish eggs such as bottarga or caviar are processed by dry salting or wet salting [21,22], and no data were found in the literature regarding the use of HPP on fish eggs.

Sea urchins have an aw of 0.98, which is considered suitable for HPP treatments, associated with a pH value (6.00) highly favorable to microbial growth, thus representing a potential risk for human health. Therefore, to obtain longer shelf life, the application of suitable technologies for keeping the product in microbiological and chemical conditions ideal for consumption is required.

This study aimed to evaluate the effect of the HPP technology on the microbial inactivation and biochemical characteristics of *P. lividus* raw gonads. Moreover, we assessed the shelf life of the gonads together with the changes in the rheological and nutritional aspects.

## 2. Materials and Methods

### 2.1. Samples Collection and Processing

Samples of *Paracentrotus lividus* (Lamarck, 1816) were collected from a rocky substrate in wild-growing areas (between 5 and 10 m depth) located at Capo Pecora, Bugerru, Italy (39°27′15.58′′ N, 8°22′43.18′′ E) in February 2015. Random specimens (*n* = 150) were harvested and carried to the laboratory in a refrigerated box (5 °C). Sea urchins were manually opened with a stainless-steel cutter. Gonads were separated from the gut, immediately stored in a water/ice bath to avoid degradation, washed with purified water, and drained in a sieve to eliminate the excess of water. Fresh gonads for each HPP treatment were merged to obtain a homogeneous sample. Finally, gonads were put in a plastic box, hot-sealed with a plastic film, and stored at 2 °C until HPP treatments.

### 2.2. HPP Processing

A FOOD PRESS 35L-600 system (Avure Technologies, JBT Group, Middletown, OH, USA) was used for the HPP process, applying two different pressures (350 and 500 MPa). Temperature (20 °C) and time (10 min) were kept constant in all experiments.

A total of 30 plastic boxes containing 100 g each of fresh raw gonads were used for each repetition (three replicates for each HPP process). Control samples not subjected to HPP treatments were stored at 5 °C.

### 2.3. Chemicals and Reagents

Hexane, ethylic ether, petroleum ether, and methanol were ultra-residue solvents of analytical grade purchased from Merck (Darmstadt, Germany). NaCl, MgSO_4_ anhydrous and Na_2_SO_4_ anhydrous, KOH, and KCl were of analytical grade (Sigma Aldrich Chemie, Darmstadt, Germany). Methanolic potash was prepared at 2 N.

The marine oil FAME mix analytical standard was purchased from Restek (Bellefonte, PA, USA). Working standard solutions were prepared by diluting the stock solution with hexane with the appropriate volume. The amino-acid mix solution was purchased from Merck (Darmstadt, Germany). Double-deionized water, with a conductivity of less than 18.2 MW, was obtained with a Milli-Q system (Millipore, Bedford, MA, USA).

### 2.4. Physical, Chemical, and Organoleptic Characteristics

For moisture determination, 5 g of homogenized samples were weighed in a crucible and dried at 105 °C until a constant weight was achieved (~24 h). Water activity (aw) was assessed by using a Thermobalance (DiniArgeo, Modena, Italy), while pH was determined with an Orion Star™ A214 pH/ISE Benchtop Meter (Thermo Fisher Scientific, Milano, Italy). The sensory quality of the raw and processed gonads was assessed according to Prato et al. [23]. Briefly, assessors (selected among regular consumers of sea urchins and trained to describe the organoleptic properties) were invited to classify the color, odor, texture, and firmness of gonads from freshly collected wild urchins and processed using a subjective scale. Score 1 was the ideal score, whereas, for texture (including firmness), the worst score was 4 (Table 1).

### 2.5. Total Lipid

The analysis of the lipid content was performed according to the Folch method. First, 0.2 g of homogenized sample was accurately weighed in an extraction thimble plus 3 mL of a CHCl_3_/CH_3_OH mixture (2/1, *v*/*v*) and 0.5 mL of a 0.88% (*w*/*v*) KCl solution. The samples were vortexed for 2 min centrifuged at 3154× *g* for 10 min.

Once the phases had been separated, 1 mL of the organic phase was placed in a previously calibrated 8 mL Pyrex glass tube. The sample was dried at room temperature and placed in a desiccator for about 1 h.

The quantitative determination of the lipid content was determined with the following formula:

g Fat = ((P/CH_3_Cl A) × CH_3_Cl B) (1)

where P is the net weight of the sample in g, CH_3_Cl A is the chloroform extract, taken and dried, in mL, and CH_3_Cl B is the chloroform used for the extraction in mL.

### 2.6. Fatty Acid

Fatty acid analyses were carried out according to Angioni et al. [4]. Briefly, 2 g of homogenized samples were accurately weighed in a screw-capped tube of 40 mL, plus 6 mL of hexane, 3 g of NaCl, and 2 g of MgSO_4_. After mixing and centrifugation, fatty acids were subjected to transesterification, adding 200 µL of alcoholic potash (KOH 2 N in MeOH) to 500 µL of the organic phase, and analyzed in GC–MS. Analysis was carried out using a gas chromatograph TRACE GC ULTRA and a Single Quad DSQ mass detector (Thermo Finnigan, Milan, Italy), equipped with a COMBI PAL autosampler (CTC Analytics, Zwingen, Switzerland) and a split/splitless injector. The column was a Varian Factor Four VFWAX (60 m × 0.25 mm i.d. × 0.25 µm film thickness; Varian, Milan, Italy). The sample (1 µL) was injected in splitless mode (1 min). Helium was the carrier gas at 1 mL/min. The mass detector was operated in electron ionization (EI) positive mode, with a solvent delay of 5.5 min. The temperature of the injector, ion source, and transfer line was set at 50, 200, and 250 °C, respectively. The oven was programmed as follows: 90 °C (1 min), raised to 160 °C (3 °C/min), then 198 °C (1 °C/min), and further to 250 °C (5 °C/min), before being held for 15 min. Peak identification was carried out comparing full mass spectra (50–550 *m*/*z*) and retention times (r.t.) from authentic standards, a homemade library, and the NIST MS spectra library.

### 2.7. Total Protein

Total protein content was analyzed according to the Kjeldahl method. An aliquot of a well-homogenized sample (0.5 g) was weighed in Speed-Digester flasks. Then, 20 mL of concentrated H_2_SO_4_ (96%), 0.5 g of sodium sulfate, and a tip of copper sulfate were added to the mineralization flask. After mineralization, the solution was left to cool, and 50 mL of H_2_O MilliQ was added. The flask was inserted into the distillation unit when the solution reached a light blue color. A concentrated solution of NaOH was added directly to the distiller and subjected to distillation until obtaining a brownish-black color. Then, 100–150 mL of sample was collected, and a known quantity of H_2_SO_4_ 0.5 N plus a few drops of methyl red were added. After distillation, quantitative analyses were conducted by titration using NaOH 0.5 N.

% protein = ((a − b) × c × 100 × K)/g sample(2)

where a is the volume of 0.5 N H_2_SO_4_ added to the collection flask in mL, b is the volume of titrant used (NaOH 0.5 N) in mL, c is the conversion factor of mL of H_2_SO_4_ 0.5 N in g of nitrogen (0.007), and K is the general nitrogen–protein conversion factor (6.25) [24].

### 2.8. Amino-Acid Analysis

An aliquot (1 g) of the homogenized sample was weighed in a 15 mL falcon tube plus 3 mL of a MeOH/H_2_O solution (20:80). The falcon was agitated for 5 min in a vortex (Falc Instrument, Treviglio, Italy) and 15 min in a rotatory wheel (F205, Falc Instrument, Treviglio, Italy); after that, it was centrifuged for 10 min at 3154× *g* and 10 °C (Centrifuge 5810 R, Eppendorf AG 22331 Hamburg, Germany). The supernatant was collected, filtered at 0.45 mm, and injected into UHPLC/MSMS for analysis.

An Agilent 1290 Infinity II UHPLC LC coupled to an Agilent 6470 Triple Quad LC–MS/MS mass detector (Agilent Technologies Italia, Milano, Italy) with a MassHunter ChemStation, was used. The column was a ZORBAX Luna Hilic (200 Å, 4.6 × 150 mm, 5 μm) at a controlled temperature of 30 °C. A binary gradient, H_2_O at 0.1% formic acid (A) and acetonitrile at 0.1% formic acid (B), was set as follows: T = 0 min, B 100%; T = 7 min, B 89%; T = 10 min, B 88%; T = 16 min, B 75%; T = 16.5 min, B 100%; post-run, 20 min. The flow was 0.6 mL/min, and the injection sample was 5 μL. The mass detector was operated in SCAN positive mode (capillary 4000 V) in the range 25–300 *m*/*z* with the following parameters: gas and curtain gas, 300 °C; gas flow, 13 L/min; curtain gas flow, 12 L/min; nebulizer, 40 psi; nozzle voltage, +1000 V. Detection was carried out in Dynamic MRM (Table 2).

### 2.9. Microbiological Analysis

Analysis was performed on fresh control raw samples and after HPP. All samples were stored in a thermostated cell at 3 °C ± 2 °C and collected after 20 and 50 days of storage. The following test methods were applied: mesophilic and psychrophilic total charge, Enterobacteriaceae, yeast and mold, sulfite-reducing anaerobic bacteria, *Pseudomonas* spp., *Salmonella* spp., and *Listeria monocytogenes* (Table 3).

### 2.10. Statistical Analysis

Analysis of variance (ANOVA) was carried out with the software XLSTAT (Addinsolf L.T.D., Version 19.4); mean comparisons of the effects of treatments were calculated by the Fisher’s least significant difference test at *p* ≤ 0.05.

## 3. Results

### 3.1. Physicochemical Analysis

Physicochemical analysis showed overlapping values for moisture among control and processed samples, ranging between 70.77 and 73.40 g/100 g, and aw averaged 0.98 among all samples (Table 4). In addition, the pH in the fresh gonads showed values of 5.84, which increased after HPP treatments and decreased during the shelf life (Table 4).

Sensory analysis showed that both treatments had similar values to the control immediately after processing, except for the texture at 350 MPa, which became smooth. In addition, after 20 days of shelf life, the treatment at 350 MPa showed a decrease in texture and firmness, as well as the emergence of a fishy odor, which became a mixture of fishy and musty after 40 days. The color changed from red-orange to dark red.

On the contrary, the treatment at 500 MPa maintained the fresh gonads’ characteristics until 20 days, with values overlapping for all sensory traits. However, after 40 days, the samples began to lose their texture and firmness, the flavor became briny/fishy, and the color changed from red-orange to pale orange (Table 5).

### 3.2. Proteins and Amino Acids

The total protein content showed an immediate decay after treatment at 350 MPa from 5.62 g/100 g (FW) to values below 4 g/100 g (FW), whereas, after HPP at 500 MPa, values overlapped with the fresh gonads and decreased after 20 days of shelf life at levels similar to 350 MPa (Table 4).

The analysis of free amino acids allowed the determination of 15 amino acids among the 17 investigated (Table 6). All essential amino acids were represented; however, the distribution in the different samples was heterogeneous. In particular, in the fresh gonads, free amino acids were mainly expressed by seven compounds, with the most represented being alanine, isoleucine, glutamine, and cysteine. After processing at 350 MPa and 500 MPa, 50% of free amino acids decreased (Table 6). During the shelf life at 350 MPa, all amino acids decreased with lower values after 40 days.

In contrast, almost all free amino acids decreased at 500 MPa after 20 days and increased after 40 days (Table 6). The sum of all free amino acids highlighted the general decrease at 350 MPa during the shelf life, whereas, at 500 MPa, there was a net increase at the end of the storage conditions.

### 3.3. Lipid Fraction

Total lipids of fresh gonad showed values ranging from 6–8 g/100 g FW, according to Angioni et al. [4]. Forty days after HPP treatment, total lipids increased in both treatments (Table 4).

A total of 29 fatty acids were identified in the sea urchin gonads. Saturated fatty acids (SAFAs) represented an average of 9.49% in the fresh samples, monounsaturated fatty acids (MUFAs) constituted 16.66%, and polyunsaturated fatty acids (PUFAs) constituted 30.49%. After HPP, total MUFAs and PUFAs remained unchanged, while SAFAs decreased at 40 days (Table 7 and Table 8). The PUFA/SAFA ratio increased at 350 MPa after 40 days, whereas it remained unchanged at 500 MPa. The total ω3 levels were two or three times higher than the ω6 levels and were not influenced by HPP treatments (Table 8).

Among PUFAs, the most represented were C20:3 n3, C20:5 n3, C20:4 n6, and C22:6 n3 (Table 7). Regarding SAFAs, the most expressed were palmitic and myristic acid; in addition, MUFAs showed a high concentration of C18:1 compounds (Table 7).

### 3.4. Microbiological Analysis

In all microbiological tests, control samples showed values consistently below 10 CFU/m^3^ (colony-forming unit).

Most of the altering microorganisms were below the suggested limit of 10 CFU/g. However, the mesophilic bacteria in the matrices at 350 MPa showed values similar to the raw control (Table 9).

Enterobacteriaceae, sulfite-reducing bacteria, *Pseudomonas* spp., yeast, and molds were below the suggested limits at all sampling times and in all matrices (Table 9). In addition, *Salmonella* spp., *Escherichia coli*, and *Listeria monocytogenes* were utterly absent.

## 4. Discussion

HPP technology consists of vacuum packing a food product and subjecting it to isostatic compression with pressure values not exceeding 700 MPa in current industrial practice. HPP microbiological activity is mainly related to extensive modifications of cellular membranes, blocking cellular functions responsible for reproduction and resulting in the most important causes of bacterial death [27]. Literature data have shown that different taxa of microorganisms and viruses work in different ways [28,29], and several studies associated with particular microorganisms have been reported [30]. On the other hand, papers dealing with the relationship between fish products and HPP treatments are limited to Atlantic species such as salmon, cod, mackerel, and sea bass [19,31,32,33].

Our experiments used a semi-industrial system at two pressure levels, 350 and 500 MPa, and a standard temperature (20 °C). The samples were packed in rectangular polypropylene (PP) trays with a capacity of 100 mL, with a closing film in PET/adhesive/PP (12/2/40 mm) and a total thickness of 54 mm.

Trials on sea urchin were successfully carried out using a model system equipped with a press with a capacity of 2 L; the samples were packaged in plastic cups and reached a shelf life of 60 days [34]. The trials assured complete microbial safety; however, no data were reported on the biochemical effect after HPP treatment and storage.

In our experiments, the microbial analysis of the working environment showed a total mesophilic count >800 CFU/m^3^ overcoming the limits considered acceptable for contamination (500 CFU/m^3^) and the presence of *Aspergillus brasiliensis*. In addition, the analysis of the water used for cleaning the gonads and the ice showed significant contamination of mesophilic bacteria. Among the two HPP treatments carried out, only 500 MPa effectively reduced contamination. In contrast, at 350 MPa, the mesophil contamination remained unchanged, with values overlapping the starting matrix. The data obtained in this study agree with previous studies on HPP applications on foods [18,30,34].

Studies on the effect of HPP on foods with a different water activity (aw) showed that lowering aw could lead to incomplete microbial inactivation [35]; the data reported in this study showed high levels of aw in all samples before and after HPP treatments allowing a suitable sanification process.

HPP treatments on fish showed an increase in pH after HPP treatments and storage for 1 week, before decreasing for more extended storage periods [20,36]. The data reported in our experiments agree with this tendency with an increase after treatment and a decrease during storage, with overlapping values in the two treatments.

In addition, sea urchins showed only relatively small pH variations before and after the treatments [20].

The fresh gonads’ physicochemical composition was in accordance with other authors on sea urchins [5,37,38]. The HPP treatments influenced the protein content during the shelf life, leading to lower values than the control samples according to similar trials on fish and crustaceans [39,40]. However, the decrease in the protein fraction was more significant after treatment at 350 MPa than at 500 MPa. These data were confirmed by analyzing the free amino-acid fraction, agreeing with Skipnes et al. (2008) [6]. At 350 MPa, the amino acids showed a higher rate of degradation associated with a characteristic fishy odor and texture modification of the gonads. As reported by Sukmanov on meat and meat products, texture modification can be related to protein denaturation [41].

On the contrary, at 500 MPa, higher levels of free amino acids were revealed. In addition, the gonads maintained the organoleptic characteristic similar to the fresh product. This fact can be ascribed to enzyme inactivation by HPP treatment, which is less effective at 350 MPa [42].

In contrast, total lipids increased after both treatments’ shelf life, following a slightly different trend.

Data on the lipid fraction of mackerel highlighted the efficiency of HPP treatments in reducing lipid damage, together with an increase in free fatty acid [43]. However, the overall free fatty-acid composition of sea urchins under HPP treatments was not affected. Indeed, a decrease in SAFAs and PUFAs, as well as an increase in MUFAs, during the shelf life at 500 MPa was detected, even if the SAFA/PUFA ratio remained unchanged. These data were in discordance with data on ragworms which showed an increase in both SAFA and MUFA, whereas data on lamb meat confirmed this behavior [39,44].

## 5. Conclusions

Although sea urchin gonads were processed in an environment contaminated by *Aspergillus brasiliensis*, the products showed no presence of this microorganism. From a microbiological perspective, the fresh sea urchin gonads showed a starting contamination of no more than 10 CFU/g. The treatments carried out at pressures of 350 MPa and 500 MPa led to obtaining samples in which microbial contamination was maintained within levels below 10 CFU/g after 40 days of storage at 4 °C.

HPP at 350 MPa resulted in a visual alteration of the texture of the fresh product immediately after processing. In addition, the fresh product’s olfactory, color, and gustatory traits were lost after 20 days. The chemical composition of the gonads confirmed organoleptic observations with a higher decrease in protein and free amino acids.

On the other hand, gonad samples stored for 40 days after treatments at 500 MPa showed a good organoleptic performance.

This paper highlighted the advantages of HPP to enhance the shelf life of sea urchin gonads. In addition, HPP caused a low modification of color and taste, preserving the nutritional and organoleptic properties of the gonads. This process, providing minimal thermal energy, can represent an effective alternative system to remove the bacterial flora and inactivate enzymes.

Moreover, to exploit the antimicrobial properties of this technology, HPP parameters should be calibrated for each specific food. Indeed, treatments at 350 MPa at 20 °C were not suitable for maintaining the organoleptic and physicochemical characteristics of unaltered sea urchins, whereas, at 500 Mpa, samples remained edible for 40 days.

On the basis of these observations, HPP could be an exciting option for enhancing sea urchins’ gonad shelf life by preserving the original organoleptic traits; more in-depth studies should be developed to obtain the ideal commercial product.

## Figures and Tables

**Table 1:** Rating system used for the evaluation of sensory characteristics of sea urchin gonads.

Score	1	2	3	4	5
Color	Red-orange	Yellow-orange	Yellow	Pale yellow	Dark brown
Flavor	Briny and sweet	Briny	Briny/fish odor	Fishy odor	Musty, yeasty
Texture	Granular	Smooth	Very smooth	No gonads	-

**Table 2:** Amino acids and LC–MS/MS-MRM *m*/*z* ions used for qualitative and quantitative analysis.

Compound Name	R.T. (min)	Precursor Ion	Product Ion	Fragmentor	Collision Energy	Cell Accelerator Voltage
Alanine	3.89	90.06	45.3	135	14	5
Histidine	6.20	156.08	83	135	14	5
Isoleucine	9.53	132.1	86.1	135	10	5
			44.2	135	18	5
Leucine	9.62	132.1	86.1	135	10	5
			30.2	135	18	5
Tryptophan	9.91	205.1	188	135	6	5
			146	135	18	5
Phenylalanine	10.02	166.09	120.1	135	14	5
Tyrosine	10.02	182.08	136.1	135	14	5
			91.1	135	18	5
Methionine	10.46	150.06	104	135	6	5
			56.1	135	18	5
Proline	11.1	116.07	70.1	135	18	5
			43.2	135	18	5
Valine	11.62	118.9	72	135	18	5
Glycine	12.64	76.04	30.3	135	18	5
Threonine	12.66	120.07	74.1	135	10	5
			56.1	135	18	5
Glutamine	13.97	147.08	130.1	135	6	5
			84.1	135	18	5
Serine	14.2	106.05	88.1	135	6	5
			42.2	135	18	5
Lysine	14.46	147.12	130.1	135	6	5
			84.1	135	18	5
Asparagine	14.73	133.06	87.1	135	12	5
Cysteine	19.47	122.03	76	135	14	5

**Table 3:** Microbiological methods used in the analysis of *P. lividus* before and after HPP processing.

Microbial Analysis	Methods
Total aerobic mesophilic charge count at 30 °C	ISO 18593:2018 + ISO 4833-1:2013
Detection and enumeration of Enterobacteriaceae	UNI ISO 21528-2:2010 + ISO 18593:2004
Beta-glucuronidase-positive *Escherichia* *coli*	UNI EN ISO 16649–3:2015/EC1:2017
Detection and enumeration of viable yeast and mold	ISO 21527-1:2008
Detection and enumeration of *Salmonella* spp.	UNI EN ISO 6579:2008
Detection and enumeration of *Listeria monocytogenes* and of *Listeria* spp.	UNI EN ISO 11290-1:2005
Detection and enumeration of psychrotrophic microorganisms	ISO 17410:2001
Detection and enumeration of *Pseudomonas* spp.	ISO 13720:2010
Count of sulfite-reducing bacteria	ISO 15213:2003

**Table 4:** Physicochemical analysis, aw, pH, total protein, and total lipid (g/100 g, FW) of fresh and HPP processed gonads of *P. lividus*.

Sample	Days	Moisture (%)	aw	pH	Total Protein	Total Lipid
		g/100 g FW			g/100 g FW	g/100 g FW
Fresh control		72.84 a	0.98 a	5.84 a	5.62 a	6.05 a
350 MPa	0	71.72 a	0.98 a	6.70 b	3.25 b	5.99 a
	20	72.30 ab	0.98 a	6.11 a	3.52 c	7.07 b
	40	73.40 b	0.99 a	5.40 c	3.76 c	13.34 c
Fresh control		71.54 a	0.98 a	5.84 a	5.94 a	8.22 d
500 MPa	0	70.77 a	0.98 a	6.50 b	5.47 a	10.31 e
	20	71.20 a	0.98 a	6.11 a	3.19 b	16.24 f
	40	71.92 a	0.99 a	5.20 c	3.20 b	13.09 c

In each column, means followed by a common letter (a–f) are not significantly different by Fisher’s least significant difference (LSD) procedure, *p* ≤ 0.05.

**Table 5:** Sensory attributes of *P. lividus* gonads, fresh, after processing, and during 40 days of storage.

Score	Day	Color	Flavor	Texture
Fresh control		1 *	1	1
350 MPa	0	1	1	2
	20	5	3	4
	40	5	4	4
500 MPa	0	1	1	1
	20	1	1	1
	40	2	1	2

* Sensory characteristics were assessed using a subjective scale of 1–5 by a trained internal panel.

**Table 6:** Free amino acid composition (mg/kg) of fresh and processed gonads of *P. lividus*.

Amino Acid	Fresh	350 MPa			Fresh	500 MPa		
		0	20	40		0	20	40
Alanine	9.75 a	9.87 a	3.54 b	1.54 c	7.75 a	6.22 a	2.87 b	7.65 a
Histidine ^e^	1.54 a	2.00 a	1.23 a	0.92 b	2.00 a	1.80 a	2.00 a	2.60 a
Isoleucine ^e^	8.36 a	1.62 b	1.45 b	0.55 c	9.86 a	10.17 a	5.21 b	12.04 a
Leucine ^e^	2.30 a	1.88 a	1.21 b	0.54 c	1.94 a	2.21a	1.59 b	2.25 a
Tryptophan ^e^	1.87 a	2.11 a	2.82 a	0.99 c	1.62 a	2.24 a	2.87 a	2.48 a
Phenylalanine ^e^	0.68 a	0.70 a	0.58 ab	0.45 b	1.41 a	1.21a	0.65 b	1.86 a
Tyrosine	3.26 a	9.60 a	4.2 a	2.68 b	3.54 a	3.75 a	3.83 a	3.92 a
Methionine ^e^	3.10 a	1.75 b	1.26 b	0.65 c	1.59 a	0.75 b	1.71 a	1.86 a
Valine ^e^	1.05 a	0.84 a	0.48 b	0.21 c	2.2 c	1.32 b	1.32 b	2.09 a
Threonine ^e^	3.19 a	3.26 b	2.62 c	1.93 d	3.26 a	2.64 a	1.25 b	3.79 a
Glutamine	4.74 a	4.60 a	4.60 a	4.25 a	3.90 a	3.91 a	2.42 b	3.45 a
Serine	3.87 a	3.91 a	2.72 b	1.70 c	2.71 a	2.06 a	2.91 a	2.75 a
Lysine ^e^	2.07 a	2.54 b	2.38 b	1.62 c	2.71 a	2.75 a	1.12 b	2.12 a
Asparagine	0.75 a	0.48 b	0.35 c	0.16 d	0.68 a	0.54 a	0.61 a	0.71 a
Cysteine	3.15 a	3.51 b	3.58 c	2.09 d	3.14 a	4.03 a	2.57 b	3.94 a
Total	49.68 a	48.67 a	33.03 b	20.28 c	48.32 a	45.60 a	32.93 b	53.51 a

In each row for each treatment, grouping means followed by a common letter (a–c) are not significantly different by Fisher’s least significant difference (LSD) procedure, *p* ≤ 0.05; ^e^ essential amino acids.

**Table 7:** Fatty-acid composition (g/100 g) of fresh and processed gonads of *P. lividus*.

Compound	T.R.	Fresh	350 MPa			Fresh	500 MPa		
			0	20	40		0	20	40
C13:0	32.89				0.13 a	0.20 a	0.25 a	0.28 a	0.12 b
C14:0	36.35	1.66 a	1.51 a	1.41 b	1.14 c	1.67 a	1.30 b	1.25 b	1.16 c
C14:1	37.45	0.86 a	0.69 a	3.38 b	1.17 a	1.12 a	1.07 a	0.59 b	1.03 a
C14:0 12-methyl (ante-iso C15)	38.04	0.42 a	0.41 a	0.37 a	0.51 a	0.24 a	0.51 b	0.37 a	0.34 a
C15:0	38.50	0.09 a	0.08 a	0.06 a	0.09 a	0.06 a	0.09 a	0.08 a	0.05 a
C15:0 13-methyl (ante-iso C16)	39.61	1.29 a	1.21 a	1.17 a	1.26 a	0.85 a	1.57 b	1.52 b	1.17 a
C16:0 14-methyl (ante-iso C17)	41.24	0.20 a	0.25 a	0.20 a	0.21 a	0.11 a	0.22 a	0.18 a	0.13 a
C16:0	42.83	3.74 a	3.59 a	3.38 a	2.27 b	5.98 a	3.55 a	4.88 a	3.75 a
C16:1 n7	43.48	0.70 a	0.52 b	0.76 a	0.46 b	0.51 a	0.57 a	0.46 a	0.59 a
C17:0	45.81	0.38 a	0.36 a	0.34 a	0.35 a	0.26 a	0.34 a	0.31 a	0.25 a
C17:1 n7	46.49	0.47 a	0.27 a	1.10 b	0.45 a	0.64 a	0.36 b	0.88 c	0.54 a
C18:0	48.78	0.82 a	0.63 b	0.50 b	0.27 c	0.44 a	0.43 a	0.49 a	0.25 b
C18:1 n9 trans	49.24	3.59 a	3.42 a	3.26 a	3.86 a	2.72 a	3.21 a	2.52 c	2.30 c
C18:1 n9 cis	49.30	3.37 a	3.46 a	5.59 b	3.23 a	8.96 a	1.84 b	2.63 c	2.07 bc
C18:2 n6	50.47	1.42 a	1.66 a	1.75 a	0.16 b	0.11 a	0.13 a	0.10 a	0.14 a
C18:2 n4	50.64	3.28 a	1.55 b	1.51 b	2.05 b	7.61 a	1.47 b	1.30 b	1.12 b
C18:3 n6	51.28	0.38 a	0.49 a	0.36 a	0.49 a	0.26 a	0.42 a	0.23 b	0.26 b
C18:3 n3	52.22	2.08 a	2.23 a	2.06 a	3.24 b	1.87 a	2.31 a	2.17 a	2.26 a
C20:0	54.28	0.36 a	0.37 a	0.37 a	0.23 a	0.18 a	0.22 a	0.24 a	0.20 a
C20:1 n9	54.71	3.12 a	3.60 a	3.40 a	1.66 b	2.40 a	2.24 a	2.24 a	9.21 b
C20:1 n7	55.94	2.28 a	1.94 a	1.20 b	1.86 a	0.86 a	0.93 a	1.41 b	1.26 b
C20:3 n6	56.60	0.39 a	1.05 b	1.02 b	1.17 b	0.64 a	1.35 b	0.88 a	0.67 a
C21:0	56.87	0.01 a	0.02 a	0.03 a	0.01 a	0.02 a	0.03 a	0.01 a	0.01 a
C20:4 n6	57.15	4.44 a	6.36 b	5.94 b	8.04 c	4.25 a	3.53 a	3.95 a	3.34 a
C20:3 n3	57.60	10.50 a	10.17 a	10.27 a	10.34 a	10.75 b	11.46 a	10.88 a	11.12 a
C20:5 n3	58.81	5.16 a	7.63 b	6.31 b	8.79 b	4.83 a	3.07 b	4.15 a	2.15 c
C22:1 n9	59.87	1.14 a	1.15 a	1.33 a	0.94 b	0.58 a	1.13 b	0.56 a	0.59 a
C22:2 n6	61.05	0.20 a	0.22 a	0.16 a	0.22 a	0.17 a	0.20 a	0.18 a	0.16 a
C22:6 n3	64.58	1.37 a	1.34 a	1.22 a	1.40 a	1.26 a	1.31 a	1.20 a	1.26 a

In each row grouping for each treatment, means followed by a common letter (a–c) are not significantly different by Fisher’s least significant difference (LSD) procedure, *p* ≤ 0.05.

**Table 8:** Total fatty-acid composition (g/100 g) of fresh and processed gonads of *P. lividus*.

		350 MPa				500 MPa		
Fatty Acid	Fresh	0	20	40	Fresh	0	20	40
Σ SAFA	8.97 a	8.43 a	7.83 ab	6.47 b	10.01 a	8.52 a	9.61 a	7.44 b
Σ MUFA	15.53 a	15.05 a	20.02 b	13.63 a	17.79 a	11.35 b	11.30 b	17.59 a
Σ PUFA	29.22 a	32.70 ab	30.60 a	35.90 b	31.75 a	25.25 b	25.04 b	22.48 b
PUFA/SAFA	3.26 a	3.88 a	3.91 a	5.55 b	3.17 a	2.97 a	2.61 a	3.02 a
Σ ω3 (%)	19.11 a	21.37 b	19.86 a	23.77 b	18.71 a	18.15 a	18.40 a	16.79 a
Σ ω6 (%)	6.83 a	9.78 b	9.23 b	10.08 b	5.43 a	5.63 a	5.34 a	4.57 a
ω3/ω6	2.80 a	2.19 a	2.15 a	2.36 a	3.45 a	3.22 a	3.45 a	3.67 a

In each row for each treatment, grouping means followed by a common letter (a, b) are not significantly different by Fisher’s least significant difference (LSD) procedure, *p* ≤ 0.05.

**Table 9:** Microbiological analysis carried out on fresh and HPP samples.

Analysis	Limits	Control	350 MPa			500 MPa		
			0	20	40	0	20	40
Total mesophilic charge at 30 °C	≤10^8^ CFU/mL	5.3 ± 0.8	4	5	5	<10	<10	<10
Total psychrophilic charge at 30 °C	≤10 CFU/g	<10	<10	<10	<10	<10	<10	<10
Enterobacteriaceae	≤10 CFU/g	<10	<10	<10	<10	<10	<10	<10
Sulfite-reducing bacteria	≤10 CFU/g	<10	<10	<10	<10	<10	<10	<10
Yeasts and molds	≤1000 CFU/g	<10	<10	<10	<10	<10	<10	<10
*Pseudomonas* spp.	≤10 CFU/g	<10	<10	<10	<10	<10	<10	<10
*Escherichia coli*	<230 MPN/100 g *	-	-	-	-	-	-	-
*Salmonella* spp.	absent/25 g *	-	-	-	-	-	-	-
*Listeria monocytogenes*	absent/25 g	-	-	-	-	-	-	-

* Reg. (UE) 2073/2005 modified by Reg. UE 1441/2007 [25,26].

## Data Availability

The datasets generated for this study are available on request to the corresponding author.
